# Incidence and risk factors of tuberculosis among 420 854 household contacts of patients with tuberculosis in the 100 Million Brazilian Cohort (2004–18): a cohort study

**DOI:** 10.1016/S1473-3099(23)00371-7

**Published:** 2024-01

**Authors:** Priscila F P S Pinto, Camila S S Teixeira, Maria Yury Ichihara, Davide Rasella, Joilda S Nery, Samila O L Sena, Elizabeth B Brickley, Maurício L Barreto, Mauro N Sanchez, Julia M Pescarini

**Affiliations:** a Centro de Integração de Dados e Conhecimentos para Saúde (Cidacs), Fundação Oswaldo Cruz, Salvador, Brazil; b Institute of Global Health (ISGlobal), Hospital Clínic—Universitat de Barcelona, Barcelona, Spain; c Instituto de Saúde Coletiva, Universidade Federal da Bahia, Salvador, Brazil; d Department of Infectious Disease Epidemiology, London School of Hygiene & Tropical Medicine, London, UK; e Núcleo de Medicina Tropical, Universidade de Brasília (UnB), Brasília, Brazil

## Abstract

**Background:**

Although household contacts of patients with tuberculosis are known to be particularly vulnerable to tuberculosis, the published evidence focused on this group at high risk within the low-income and middle-income country context remains sparse. Using nationwide data from Brazil, we aimed to estimate the incidence and investigate the socioeconomic and clinical determinants of tuberculosis in a cohort of contacts of tuberculosis patients.

**Methods:**

In this cohort study, we linked individual socioeconomic and demographic data from the 100 Million Brazilian Cohort to mortality data and tuberculosis registries, identified contacts of tuberculosis index patients diagnosed from Jan 1, 2004 to Dec 31, 2018, and followed up the contacts until the contact's subsequent tuberculosis diagnosis, the contact's death, or Dec 31, 2018. We investigated factors associated with active tuberculosis using multilevel Poisson regressions, allowing for municipality-level and household-level random effects.

**Findings:**

We studied 420 854 household contacts of 137 131 tuberculosis index patients. During the 15 years of follow-up (median 4·4 years [IQR 1·9–7·6]), we detected 8953 contacts with tuberculosis. The tuberculosis incidence among contacts was 427·8 per 100 000 person-years at risk (95% CI 419·1–436·8), 16-times higher than the incidence in the general population (26·2 [26·1–26·3]) and the risk was prolonged. Tuberculosis incidence was associated with the index patient being preschool aged (<5 years; adjusted risk ratio 4·15 [95% CI 3·26–5·28]) or having pulmonary tuberculosis (2·84 [2·55–3·17]).

**Interpretation:**

The high and sustained risk of tuberculosis among contacts reinforces the need to systematically expand and strengthen contact tracing and preventive treatment policies in Brazil in order to achieve national and international targets for tuberculosis elimination.

**Funding:**

Wellcome Trust and Brazilian Ministry of Health.

## Introduction

Tuberculosis is an infectious respiratory disease, for which transmission in high-burden settings is primarily reduced by addressing the underlying social determinants, achieving rapid diagnoses and effective treatments for new tuberculosis, and actively and systematically screening contacts.^1^ In high-income countries with a low tuberculosis burden, screening of active and latent tuberculosis among contacts is routinely performed as part of tuberculosis programmes.^2^ However, in low-income and middle-income countries (LMICs), screening of contacts has yet to be carried out systematically.^3^ In the high-burden country of Brazil, where the incidence of tuberculosis was 32·0 per 100 000 people in 2022,^1^, ^3^ the Ministry of Health recommends that all contacts should be evaluated. However, in 2021, only 69·1% of high-priority contacts (eg, contacts of index patients with laboratory-confirmed pulmonary tuberculosis) were examined.^3^

Although household contacts of patients with tuberculosis are known to be particularly vulnerable to infection themselves,^4^ the published evidence focused on this group at high risk within the LMIC context remains scarce**.** Previous research from Peru and Ethiopia suggests that household contacts experience an 8–10-times higher incidence of tuberculosis than the general population of those countries.^5^, ^6^ Furthermore, a small body of evidence on risk factors for tuberculosis among contacts in LMICs suggests increased risks for contacts who are young, male, have comorbidities (eg, HIV, undernutrition, or diabetes), or who reside with an index patient with pulmonary tuberculosis.^5^, ^7^, ^8^, ^9^ Nevertheless, the role of poverty, overcrowding, and other socioeconomic determinants of tuberculosis among contacts remains inadequately studied to date.

Using nationwide data from Brazil, we linked socioeconomic and tuberculosis data with the aim of estimating the incidence of tuberculosis in a cohort of household contacts of low-income tuberculosis patients; and to investigate the clinical, geographical, and socioeconomic factors associated with tuberculosis among contacts.

**Research in context**

**Evidence before this study**

We searched MEDLINE and SCOPUS for studies in English, Portuguese, or Spanish using the terms “mycobacterium tuberculosis”, “tuberculosis pulmonary”, “contact tracing”, “household*”, “family contact*”, “household contact*”, “childhood contact*”, “disease transmission, infectious”, “household transmission”,” contact*”, “contact screen*”, “contact investigation”, “close contact*”, and “contact examination”. We found ten cohort studies that investigated the incidence or factors associated with active tuberculosis among household contacts in low-income and middle-income countries from Jan 1, 2012 to March 31, 2023, of which only two used administrative data from national tuberculosis registry systems. The study with the largest population included contacts of up to 14 000 individuals for up to 5 years. Two studies, conducted in Ethiopia and Peru, reported that the incidence of tuberculosis was 8–10-times higher among household contacts than the general population in those countries. Among the ten studies, of which two were conducted in Brazil, higher tuberculosis incidence was found among contacts who were male (in one study), of younger age (ie, younger than 5 years; two studies), 15 years or older (two studies), had close or intimate contact or intense exposure with the tuberculosis index patient (two studies), experienced undernutrition (one study), had HIV infection (six studies), had diabetes (one study), or were previously infected with tuberculosis (one study). Pulmonary tuberculosis, high bacillary load, and presence of cavitation on chest x-ray were the index patient characteristics reported to be associated with tuberculosis among household contacts (two studies).

**Added value of this study**

This is the first study to use a cohort of household contacts who were identified and followed up through the linkage of administrative data to estimate tuberculosis incidence and its risk factors. Using data from 420 854 low-income household contacts followed up for 15 years in a high-burden setting for tuberculosis, we observed prolonged risk of tuberculosis detection among household contacts that was approximately 16-times higher than those of non-cohabitants. Relative to non-cohabitants, preschool-aged children (younger than 5 years) who were a contact of a patient with tuberculosis experienced an incidence of tuberculosis that was 62-times higher, with the most pronounced risk in the first six months after the detection of the index patient with tuberculosis. As tuberculosis incidence in preschool-aged children remained relatively stable thereafter, we hypothesise that timely contact tracing and preventive treatment might contribute to tuberculosis prevention in this age group. The tuberculosis incidence among contacts overall was also higher when contacts were socially or economically disadvantaged (ie, of Black, Pardo, or indigenous race or ethnicity, or living in poor housing conditions) and when the index patients were younger than five years or had pulmonary tuberculosis. Finally, poor quality of municipal health services was associated with higher tuberculosis among household contacts younger than 5 years and cohabitants who were detected more than three months later than the index patient.

**Implications of all the available evidence**

The exceptionally high tuberculosis incidence among contacts in our cohort suggests that greater efforts should be directed towards active and systematic evaluation of contacts in Brazil, especially adolescents, young adults, more socially vulnerable household contacts, and cohabitants from index patients younger than 5 years or with pulmonary tuberculosis. As preventive tuberculosis treatment is key to mitigate progression to tuberculosis disease, strengthening and expanding contact tracing could contribute to tuberculosis prevention and care in Brazil and other countries with a high burden of tuberculosis.

## Methods

### Study design and data sources

We followed up a cohort of household contacts (also termed cohabitants) of patients with tuberculosis obtained through the linkage of individual demographic and socioeconomic data from individuals registered in the 100 Million Brazilian Cohort (100MCohort) from 2004 to 2018,^10^ and information from nationwide tuberculosis registries (Sistema de Informação de Agravos de Notificação for tuberculosis [SINAN-TB]) from 2004 to 2018, and mortality data (Sistema de Informação sobre Mortalidade [SIM]) from 2001 to 2018. The 100MCohort is a cohort of individuals on low-income applying for social programmes in Brazil registered in the National Registry for Social Programmes (CadÚnico; appendix p 2).^10^ Linkage was performed using Centro de Integração de Dados e Conhecimentos para Saúde Record Linkage (CIDACS-RL), which uses a two-step strategy. The first step is a fully deterministic linkage based on five identifying variables (ie, name, mother's name, sex, date of birth, and the municipality of residence). The second step is non-deterministic and uses the same five variables to produce a similarity score; matched registries are based on scores of optimal sensitivity and specificity thresholds.^10^ Linkage accuracy between tuberculosis registries and the 100MCohort was measured in terms of sensitivity (94·6%) and specificity (93·6%) calculated based on false or true links between the two databases (appendix p 3).^11^ Linkage accuracy between mortality registries and the 100MCohort was similarly calculated by year: sensitivity ranged between 97·8% and 100·0% and specificity between 96·6% and 99·9% (appendix p 4). After linkage, the dataset was de-identified, and the researcher accessed the data through a virtual private network in a data safe haven without access to the internet.^10^

From the 100MCohort, we extracted all the socioeconomic and demographic variables at the individual and household level for the index patient with tuberculosis and the cohabitants (ie, age, sex, education, self-identified race or ethnicity, Brazilian region and area of residence [rural or urban], household density, housing materials, water supply, sewage, lighting, and garbage disposal), as well as information on which individuals were living in the same household at the time of registration (ie, identified through a family code). For individuals younger than 16 years, we used the education of the oldest member of the household as a proxy for the education of the household head. From SINAN, we extracted the date of diagnosis and clinical classification (pulmonary or extrapulmonary tuberculosis) from patients diagnosed with tuberculosis. People diagnosed with pulmonary plus extrapulmonary tuberculosis were classified as pulmonary tuberculosis. From SIM, we extracted the date of death for those who died during the study period. From the database of a previous study, we extracted information on the performance of tuberculosis indicators in the index patient's municipality of residence.^12^

This study was reported according to RECORD. The study was approved by the ethics committees of the Instituto Gonçalo Muniz–Oswaldo Cruz Foundation (1.612.302 in 2016), Salvador, Brazil.

### Study setting and participants

This study included all cohabitants who applied to the 100MCohort between Jan 1, 2004 and Dec 31, 2018. We excluded individuals who were aged 100 years or older, diagnosed with tuberculosis before or on the same day of the application to the 100MCohort, or experiencing homelessness (due to the impossibility of identifying their cohabitants using CadÚnico). In each household, we defined the first new patient with tuberculosis detected as the index patient and the individuals living in the same household as the contacts. Of note, this study did not assume a direct chain of transmission between the index patient and subsequent tuberculosis among contacts. In the 225 families with more than one individual diagnosed on the same date, both were considered index patients, but one was chosen randomly for the attribution of index patient clinical characteristics.

### Outcome

We focused our analysis on active tuberculosis, as the latent tuberculosis infection (LTBI) notification system was only implemented in 2014 with the corresponding electronic information system introduced gradually since 2018.^3^ The primary endpoint was the detection of tuberculosis among cohabitants following the diagnosis of the index patient in the overall population of household contacts. The secondary endpoint was the detection of tuberculosis among contacts aged 5 years or younger. All cohabitants were followed up from the date of diagnosis of the index patient until the contact's subsequent tuberculosis diagnosis, the contact's death, or by Dec 31, 2018, whichever date came first. In the subanalysis of children aged younger than 5 years, children were censored on their fifth birthday.

### Exposures

Key potential risk factors included the individual-level and household-level characteristics of both the cohabitant and the index patient as well as individual-level clinical characteristics of the index patient. As tuberculosis contact tracing might reflect differing policies and budgets across municipalities and states, we further classified individuals by the performance of tuberculosis indicators in the index patient's municipality of residence. This composite variable reflects the quality of the health-care services provided for patients with tuberculosis based on six operational indicators: laboratory confirmation, contact tracing, HIV testing, directly observed therapy use, treatment dropout, and cure rate. Under this classification, Group A represents municipalities with the highest quality performance for tuberculosis indicators; Group B represents municipalities with medium quality performance; and Group C represents municipalities with the lowest quality performance.^12^

### Statistical analysis

We estimated the incidence and cumulative hazard of tuberculosis per 100 000 cohabitants at risk (person-years at risk). Since 2004, the Brazilian Ministry of Health has recommended tuberculosis preventive treatment for children younger than 5 years with LTBI and, in 2010, tuberculosis preventive treatment was expanded to contacts older than 10 years.^13^ In 2014, tuberculosis preventive treatment was temporarily extended to children without LTBI testing given the shortage of these tests in Brazil.^14^ Therefore, we also estimated the incidence and cumulative hazard of tuberculosis stratified by age group of the contact, tuberculosis classification (ie, pulmonary and extrapulmonary tuberculosis), and municipality-level indicators of tuberculosis performance.

**Figure 1:**
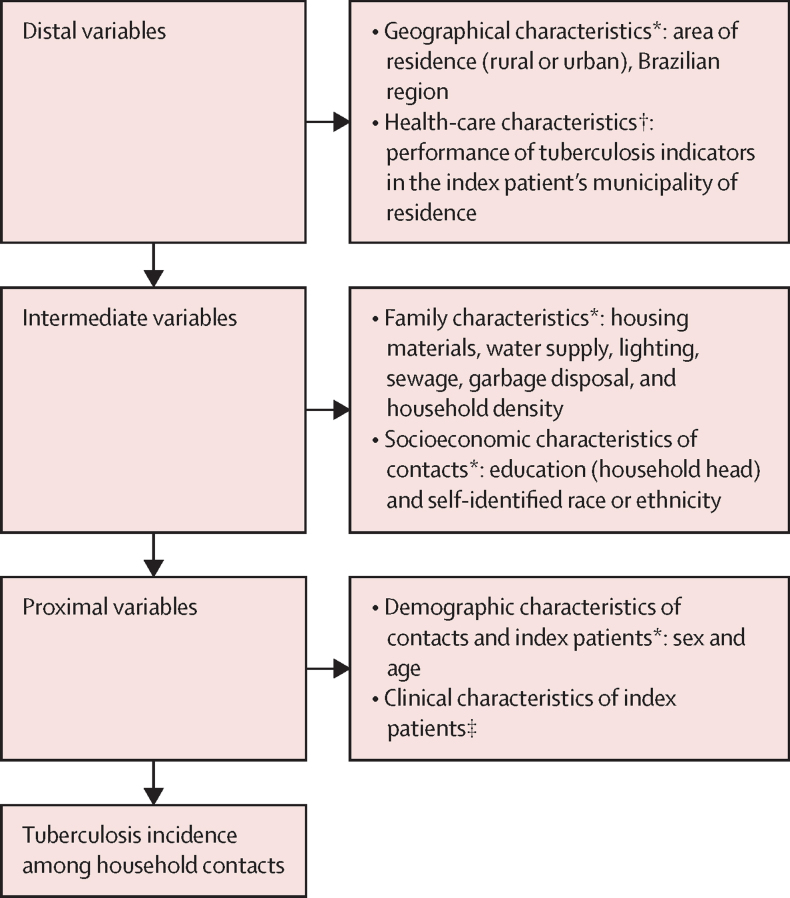
Hierarchical conceptual framework for adjusted multilevel Poisson regression model CadÚnico=Brazil National Registry for Social Programmes (Cadastro Único). SINAN-TB=Sistema de Informação de Agravos de Notificação for tuberculosis database. *Information from CadÚnico. †Information from the database of a previous study.^12^ ‡Information from SINAN-TB.

To investigate the factors associated with tuberculosis among contacts, we estimated the crude and adjusted risk ratios (RRs) using multilevel mixed-effects Poisson regressions, allowing for the municipality-specific and household-specific random effects. As a sensitivity analysis, we repeated the analysis but excluded contacts who were diagnosed within 3 months of the detection of the index patient as they could be considered co-prevalent patients.^2^, ^15^, ^16^ For each multilevel Poisson regression model the intraclass correlation coefficient (ICC) was estimated. We performed a sub-analysis for children younger than 5 years using a multivariable Poisson regression. Of note, we did not perform a multilevel regression due to the low number of young children per household. Adjusted models were built using a hierarchical analysis, inserting the variables through the full model strategy based on a conceptual framework^17^ that includes: first, geographical characteristics and municipality-level indicators of tuberculosis performance as distal variables; second, household-level socioeconomic characteristics and their proxies as intermediate variables; and third, individual-level demographic characteristics of cohabitants and index patients and individual-level clinical characteristics of index patients as proximal variables (figure 1).

To investigate if risk factors for tuberculosis among cohabitants depended on the quality of local tuberculosis programmes, we conducted subgroup analyses stratified across the three groups representing municipality-level indicators of tuberculosis performance.

For the all-age groups and the subgroup of contacts younger than 5 years, we estimated the percent attributable risk (%AR) of having tuberculosis in exposed individuals as the following: %AR_exp_=(Incidence_exp_ − Incidence_nexp_) × 100/Incidence_exp_.^18^ To do that, we estimated the tuberculosis incidence in the overall 100MCohort population and used it as a proxy of the tuberculosis incidence in the unexposed population.

All analyses were performed using Stata, version 15.1.

### Role of the funding source

The funding institutions of the study had no role in the study design, data collection, data analysis, data interpretation, or writing of the report.

## Results

**Figure 2:**
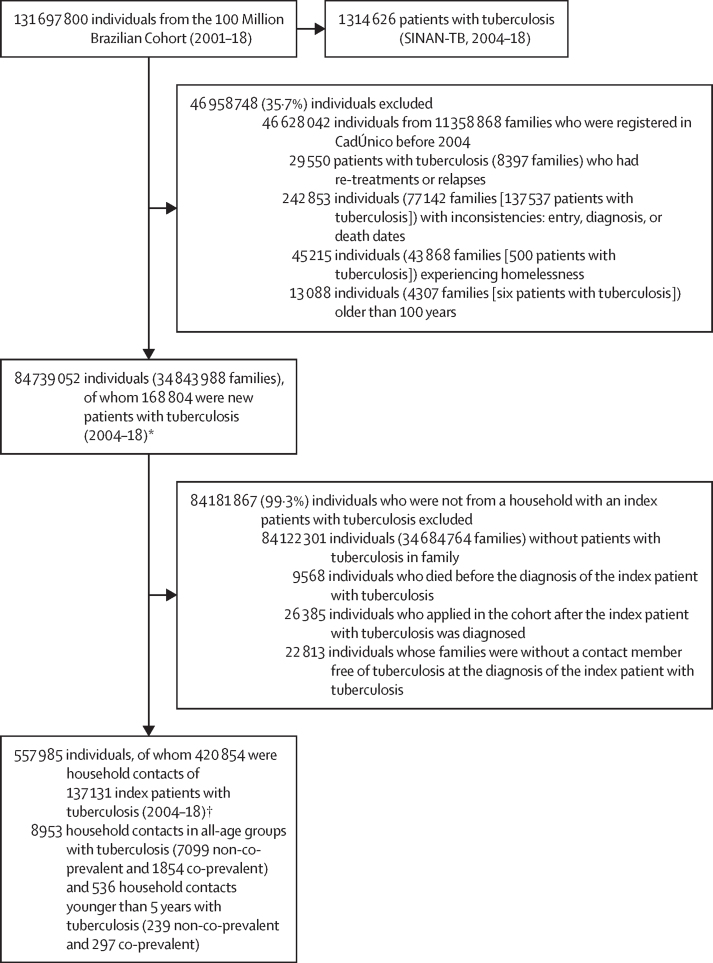
Selection of study population CadÚnico=Brazil National Registry for Social Programmes (Cadastro Único). SINAN-TB=Sistema de Informação de Agravos de Notificação for tuberculosis database. *Population used as a proxy of non-exposed to calculate the percent attributable risk of being a household contact. †Population used to calculate tuberculosis incidence among contacts and its associated factors.

Of the 84 739 052 individuals included in the 100MCohort between Jan 1, 2004 and Dec 31, 2018, we identified 168 804 new patients with tuberculosis with an incidence of 26·2 per 100 000 person-years at risk (95% CI 26·1–26·3; figure 2, appendix p 5). After excluding the 84 181 867 (99·3%) of 84 739 052 individuals who did not share a household with an index patient with tuberculosis, we studied 420 854 household contacts of 137 131 index patients with tuberculosis (figure 2). During the 15 years of follow-up (median 4·4 years [IQR 1·9–7·6]), tuberculosis was detected among 8953 (2·1% [95% CI 2·1–2·2]) of 420 854 cohabitants (table 1). The mean incidence of tuberculosis among household contacts was 427·8 per 100 000 person-years at risk (95% CI 419·1–436·8) versus 26·2 (26·1–26·3) in the 100MCohort overall (table 1, appendix p 5).

**Table 1:** Descriptive analysis and incidence of tuberculosis among household contacts

			**Contacts**	**Contacts diagnosed with tuberculosis**	**Persons-years at risk**	**Incidence per 100 000 persons-years (95% CI)**
Total			420 854 (100·0%)	8953 (100·0%)	2 092 550·5	427·8 (419·1–436·8)
Characteristics of the index patient						
	Sex					
		Female	175 799 (41·8%)	4331 (48·4%)	925 896·6	467·8 (454·0–481·9)
		Male	245 055 (58·2%)	4622 (51·6%)	1 166 653·9	396·2 (384·2–407·8)
	Age (years)					
		<5	4432 (1·1%)	178 (2·0%)	27 701·9	642·6 (554·8–744·2)
		5–14	17 109 (4·1%)	556 (6·2%)	94 526·0	588·2 (541·3–639·2)
		15–19	44 943 (10·7%)	1432 (16·0%)	200 104·7	715·6 (679·5–753·7)
		20–59	326 890 (77·7%)	6445 (72·0%)	1 641 434·1	392·6 (383·2–402·3)
		≥60	27 480 (6·5%)	342 (3·8%)	128 783·8	265·6 (238·8–295·2)
Clinical classification of tuberculosis						
		Extrapulmonary	54 786 (13·0%)	485 (5·4%)	277 708·8	174·6 (159·8–190·9)
		Pulmonary	366 068 (87·0%)	8468 (94·6%)	1 814 841·8	466·6 (456·8–476·6)
Characteristics of the contact						
	Sex					
		Female	226 740 (53·9%)	4392 (49·1%)	1 122 218·2	391·4 (380·0–403·1)
		Male	194 114 (46·1%)	4561 (50·9%)	970 332·3	470·0 (456·6–483·9)
	Age (years)					
		<5	32 273 (7·7%)	536 (6·0 %)	211 017·2	254·0 (233·4–276·4)
		5–14	124 599 (29·6%)	2107 (23·5%)	698 774·9	301·5 (288·9–314·7)
		15–19	58 767 (14·0%)	1831 (20·5%)	266 887·0	686·0 (655·3–718·2)
		20–59	183 604 (43·6%)	4185 (46·7%)	830 478·6	503·9 (488·9–519·4)
		≥60	21 611 (5·1%)	294 (3·3%)	85 392·8	344·3 (307·1–386·0)
	Race or ethnicity					
		White	114 115 (27·1%)	2342 (26·2%)	560 621·5	417·7 (401·2–435·0)
		Black	43 394 (10·3%)	1148 (12·8%)	223 124·5	514·5 (485·6–545·1)
		Asian	1163 (0·3%)	15 (0·2%)	5313·8	282·3 (170·2–468·2)
		Pardo	245 970 (58·4%)	4999 (55·8%)	1 227 834·6	407·1 (396·0–418·6)
		Indigenous	8194 (1·9%)	267 (3·0%)	38 623·7	691·3 (613·1–779·4)
		Missing	8018 (1·9%)	182 (2·0%)	−	−
	Education (years of study)					
		>9	52 495 (12·5%)	968 (10·8%)	224 462·2	431·3 (404·9–459·3)
		4–9	123 770 (29·4%)	2805 (31·3%)	614 153·5	456·7 (440·1–473·9)
		<4	143 621 (34·1%)	3156 (35·3%)	715 714·6	440·9 (425·8–456·6)
		Illiterate	55 755 (13·2%)	1118 (12·5%)	294 343·0	379·8 (358·2–402·8)
		Missing	45 213 (10·7%)	906 (10·1%)	..	..
Household characteristics						
	Area of residence					
		Urban	364 990 (86·7%)	7930 (88·6%)	1 797 514·9	441·2 (431·6–451·0)
		Rural	55 449 (13·2%)	1006 (11·2%)	292 831·0	343·5 (322·9–365·4)
		Missing	415 (0·1%)	17 (0·2%)	..	..
	Brazilian region					
		North	58 061 (13·8%)	1137 (12·7%)	183 448·8	401·1 (378·5–425·1)
		Northeast	118 350 (28·1%)	1957 (21·9%)	632 133·7	309·6 (296·2–323·6)
		Midwest	19 623 (4·7%)	384 (4·3%)	93 295·2	411·6 (372·4–454·9)
		Southeast	183 454 (43·6%)	4554 (50·9%)	874 474·1	520·8 (505·9–536·1)
		South	41 362 (9·8%)	921 (10·3%)	209 170·1	440·3 (412·8–469·7)
		Missing	4 (<0·1%)	0	..	..
Household density (residents per room)						
		<3·0	362 561 (86·1%)	7570 (84·6%)	1 819 997·5	415·9 (406·7–425·4)
		≥3·0	54 747 (13·0%)	1305 (14·6%)	260 362·4	501·2 (474·7–529·2)
		Missing	3546 (0·8%)	78 (0·9%)	..	..
	House material					
		Brick	316 940 (75·3%)	6663 (74·4%)	1 558 111·2	427·6 (417·5–438·0)
		Wood	62 934 (15·0%)	1482 (16·6%)	319 798·8	463·4 (440·4–487·6)
		Other materials (taipa*)	37 434 (8·9%)	730 (8·2%)	202 440·4	360·6 (335·4–387·7)
		Missing	3546 (0·8%)	78 (0·9%)	..	..
	Water supply					
		Public network	316 937 (75·3%)	6862 (76·6%)	1 562 627·0	439·1 (428·9–449·6)
		Well, spring, or cistern	100 368 (23·8%)	2013 (22·5%)	517 727·6	388·8 (372·2–406·2)
		Missing	3549 (0·8%)	78 (0·9%)	..	..
	Sewage					
		Public network	225 299 (53·5%)	5055 (56·5%)	1 093 389·8	462·3 (449·7–475·2)
		Septic tank	50 329 (12·0%)	905 (10·1%)	255 565·6	354·1 (331·8–377·9)
		Rudimentary cesspit, ditch, or another	138 475 (32·9%)	2865 (32·0%)	721 857·3	396·9 (382·6–411·7)
		Missing	6751 (1·6%)	128 (1·4%)	..	..
	Lighting					
		Electricity with household meter	304 537 (72·4%)	6106 (68·2%)	1 521 755·0	401·2 (391·3–411·4)
		Electricity with collective meter (one meter for multiple houses)	26 355 (6·3%)	623 (7·0%)	124 910·4	498·7 (461·1–539·5)
		Electricity without meter (irregular source)	48 622 (11·6%)	1268 (14·2%)	238 718·1	531·2 (502·7–561·2)
		No electricity (eg, lamp or candle)	37 790 (9·0%)	878 (9·8%)	194 962·0	450·3 (421·5–481·1)
		Missing	3550 (0·8%)	78 (0·9%)	..	..
	Garbage disposal					
		Public collection	348 495 (82·8%)	7513 (83·9%)	171 4105·2	438·3 (428·5–448·3)
		Burned, buried, or another	68 810 (16·4%)	1362 (15·2%)	366 249·3	371·9 (352·6–392·2)
		Missing	3549 (0·8%)	78 (0·9%)	..	..
Performance of tuberculosis indicators in the index patient's municipality†						
		Group A (highest)	133 511 (31·7%)	2896 (32·3%)	670 675·3	431·8 (416·4–417·8)
		Group B (medium)	91 609 (21·8%)	1720 (19·2%)	460 774·1	373·3 (356·1–391·3)
		Group C (lowest)	177 589 (42·2%)	4054 (45·3%)	860 685·5	471·0 (456·7–485·7)
		Missing	18 145 (4·3%)	283 (3·2%)	..	..

* Taipa is a construction method that consists of using clay and wood to build houses.

† Group A represents municipalities with the highest quality performance for tuberculosis indicators; Group B represents municipalities with medium quality performance for tuberculosis indicators; Group C represents municipalities with the lowest quality performance for tuberculosis indicators.

**Figure 3:**
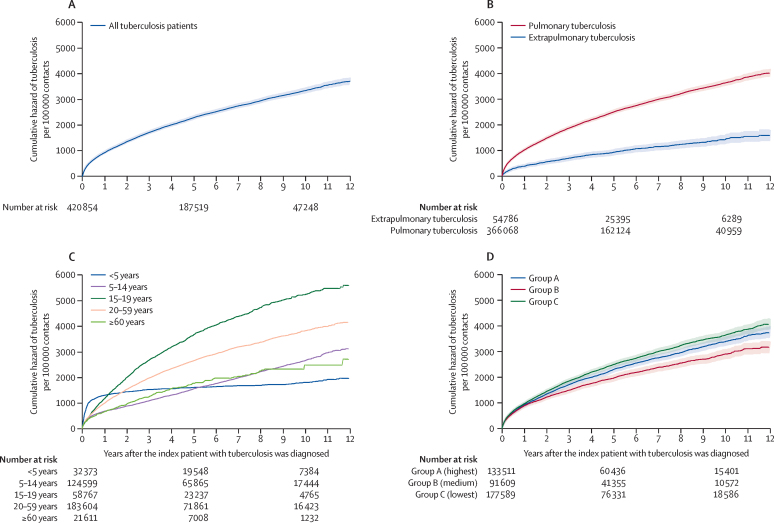
Tuberculosis cumulative hazard among household contacts overall (A), and by index patient clinical classification (B), age of the contact (C), and performance of tuberculosis indicators in the municipality (D) Follow-up time limited to 12 years to facilitate visualisation. 95% CIs for age-specific risks for the final years of follow-up in the older age category were too large and therefore not computed as they would compromise the graph visualisation. Group A represents municipalities with the highest quality performance for tuberculosis indicators; Group B represents municipalities with medium quality performance for tuberculosis indicators; Group C represents municipalities with the lowest quality performance for tuberculosis indicators.

The plurality of cohabitants was female (226 740 [53·9%] of 420 854), aged 20–59 years (183 604 [43·6%]) and of Pardo race or ethnicity (245 970 [58·4%]; table 1). In 2338 (26·1%) of 8953 households with tuberculosis among contacts, more than one cohabitant was diagnosed after the index patient. The tuberculosis incidence was higher among contacts who self-identified as indigenous (691·3 per 100 000 person-years at risk [95% CI 613·1–779·4]), were aged 15–19 years (686·0 [655·3–718·2]), and had an index patient with tuberculosis aged 15–19 years (715·6 [679·5–753·7]; table 1). The tuberculosis incidence among household contacts was highest in the first year after the diagnosis of the index patient (953·3 per 100 000 person-years at risk [95% CI 923·2–984·4]; appendix p 6). The cumulative hazard curves of tuberculosis indicate that there was a prolonged risk of subsequent tuberculosis in the exposed household contacts mainly among cohabitants aged 15–59 years and whose index patient was diagnosed with pulmonary tuberculosis (figure 3).

When the index patient was younger than 5 years (adjusted RR 4·15 [95% CI 3·26–5·28]) or had pulmonary tuberculosis (2·84 [2·55–3·17]), there was a higher risk of tuberculosis among contacts. Tuberculosis among cohabitants was also associated with race and ethnicity (ie, indigenous, Pardo, or Black) and poor housing conditions (ie, houses constructed with wood, lacking electricity, or with irregular electricity sources; table 2). As compared with the 1854 (20·7%) of 8953 co-prevalent patients, contacts with non-co-prevalent tuberculosis were more likely to reside in municipalities with the lowest quality performance for tuberculosis indicators (appendix p 7).

**Table 2:** Adjusted multilevel Poisson regression analysis of the association of distal, intermediate, and proximal variables with tuberculosis among household contacts

		Adjusted RR*(95% CI)
**Distal variables**†		
Area of residence		
	Rural	1 (ref)
	Urban	1·05 (0·96–1·16)
Brazilian region		
	North	1 (ref)
	Northeast	0·79 (0·69–0·89)
	Midwest	1·05 (0·87–1·26)
	Southeast	1·20 (1·07–1·36)
	South	1·19 (1·02–1·38)
Performance of tuberculosis indicators in the index patient's municipality‡		
	Group A (highest)	1 (ref)
	Group B (medium)	0·96 (0·88–1·05)
	Group C (lowest)	1·06 (0·96–1·16)
**Intermediate variables**§		
Education (years of study)		
	>9	1 (ref)
	4–9	1·03 (0·95–1·11)
	<4	1·01 (0·93–1·10)
	Illiterate	0·95 (0·86–1·06)
Race or ethnicity		
	White	1 (ref)
	Asian	0·70 (0·38–1·27)
	Pardo	1·11 (1·05–1·19)
	Black	1·27 (1·17–1·39)
	Indigenous	2·19 (1·77–2·70)
House material		
	Brick	1 (ref)
	Wood	1·12 (1·03–1·23)
	Other materials (taipa¶)	0·98 (0·87–1·09)
Water supply		
	Public network	1 (ref)
	Well, spring, or cistern	0·92 (0·85–1·00)
Sewage		
	Public network	1 (ref)
	Septic tank	0·99 (0·90–1·10)
	Rudimentary cesspit, ditch, or another	1·04 (0·97–1·12)
Lighting		
	Electricity with household meter	1 (ref)
	Electricity with collective meter (one meter for multiple households)	1·08 (0·97–1·20)
	Electricity without meter (irregular source)	1·13 (1·04–1·22)
	No electricity (eg, lamp or candle)	1·13 (1·02–1·25)
Garbage disposal		
	Public collection	1 (ref)
	Burned, buried, or another	1·01 (0·92–1·12)
Household density (residents per room)		
	<3	1 (ref)
	≥3	0·96 (0·89–1·04)
**Proximal variables**‖		
Sex of the subsequent patient among contacts		
	Female	1 (ref)
	Male	1·24 (1·18–1·30)
Age (years) of the subsequent patient among contacts		
	<5	1 (ref)
	5–14	0·83 (0·74–0·92)
	15–19	1·58 (1·41–1·77)
	20–59	1·25 (1·13–1·39)
	≥60	0·90 (0·76–1·06)
Sex of the index patient		
	Male	1 (ref)
	Female	1·40 (1·32–1·47)
Age (years) of the index patient		
	≥60	1 (ref)
	20–59	1·40 (1·22–1·60)
	15–19	2·17 (1·87–2·51)
	5–14	2·70 (2·27–3·21)
	<5	4·15 (3·26–5·28)
Clinical classification of tuberculosis index patient		
	Extrapulmonary	1 (ref)
	Pulmonary	2·84 (2·55–3·17)

RR=risk ratio.

* Considering municipality-level and household-level as random effects.

† Distal model (n=350 119) RR was adjusted for area of residence and Brazilian region, and performance of tuberculosis indicators in the index patient's municipality of residence (distal variables).

‡ Group A represents municipalities with the highest quality performance for tuberculosis indicators; Group B represents municipalities with medium quality performance for tuberculosis indicators; Group C represents municipalities with the lowest quality performance for tuberculosis indicators.

§ Intermediate model (n=350 119) RR was adjusted for education, self-identified race or ethnicity, house material, water supply, sewage, lighting, garbage disposal, household density (intermediate variables) plus area of residence and Brazilian region, and performance of tuberculosis indicators in the index patient's municipality of residence (distal variables).

¶ Taipa is a construction method that consists of using clay and wood to build houses.

‖ Proximal model (n=350 119) RR was adjusted for sex and age of subsequent patient among contacts, sex and age of tuberculosis index patient, and clinical classification of the tuberculosis index patient (proximal variables), plus education, race or ethnicity, house material, water supply, sewage, lighting, garbage disposal, household density (intermediate variables), plus area of residence and Brazilian region, and performance of tuberculosis indicators in the index patient's municipality of residence (distal variables).

Among the 32 273 household contacts younger than 5 years, 536 (1·7% [95% CI 1·5–1·8]) were diagnosed with tuberculosis during follow-up. The incidence of tuberculosis among cohabitants younger than 5 years was 254·0 per 100 000 person-years at risk (95% CI 233·4–276·4; table 1; appendix p 9) versus 4·1 per 100 000 (4·0–4·2) among children younger than 5 years in the 100MCohort overall (appendix p 5). The most important period for detecting tuberculosis among cohabitants younger than 5 years was in the first 6 months following the index patient's diagnosis, when 68·1% of diagnoses among preschool-aged children (<5 years) occurred. After this, the incidence remained stable in this group (figure 3). For preschool-aged contacts, the main risk factors for tuberculosis were having an index patient younger than 5 years, indigenous race or ethnicity, houses constructed out of wood, and the lowest quality health service performance for tuberculosis indicators (appendix p 11). For the 239 (44·6%) of 536 children younger than 5 years with non-co-prevalent tuberculosis, only the characteristics of the index patient were associated with tuberculosis (appendix p 13).

In the analyses stratified by the municipality performance for tuberculosis indicators, we found in all the three groups a higher risk of tuberculosis among contacts who were of indigenous, Pardo, or Black race or ethnicity, lived in poor housing conditions, and had an index patient younger than 5 years or who was diagnosed with pulmonary tuberculosis (appendix pp 15–18). Finally, we estimated that the %AR_exp_ of being a cohabitant and developing tuberculosis was 93·9% for the all-age groups and 98·4% for children younger than 5 years (appendix p 18).

The ICC values for the multilevel Poisson regression analysis are presented in the appendix p 18.

## Discussion

Our analysis of low-income contacts in the 100MCohort suggests that after the initial diagnosis of tuberculosis in a household, cohabitants experienced a high and sustained risk of tuberculosis, which was 16-times higher than the incidence in the 100MCohort non-cohabitant population. For children younger than 5 years, the incidence was 62-times higher among cohabitants than non-cohabitants. Contacts of an index patient younger than 5 years or with pulmonary tuberculosis and cohabitants experiencing indicators of social vulnerability in Brazil (ie, Black, Pardo, or indigenous race or ethnicity, and living in poor housing conditions) had a higher risk of tuberculosis.

A key limitation of this investigation is that important confounding variables (eg, intensity of exposure,^19^ LTBI testing results, use of tuberculosis preventive treatment, and HIV infection status^20^) were not available in our study. Therefore, it is impossible to determine whether household contacts may have had tuberculosis via transmission from the index patient, from an exposure outside the home, or from reactivation of LTBI. It should be noted that in high-burden settings such as Brazil, tuberculosis transmission is more likely to occur outside the household.^21^ However, the high rates of tuberculosis in contacts and the high attributable risk for being a contact found in our study could indicate an intense tuberculosis transmission within households.

The higher incidence of tuberculosis among contacts than in the general population, a finding aligned with the observations from other studies in LMICs,^5^, ^6^, ^7^, ^15^, ^22^ might result from increased awareness of the disease and consequent diagnosis in cohabitants of the index patient with tuberculosis, as well as from the clustering of risk factors within the households. Additionally, the detection of tuberculosis in the index patient might have precipitated tuberculosis screening among the contacts. Notably, a previous study using a similar approach to study leprosy in the 100MCohort also identified a high incidence of new leprosy case detections among household contacts,^23^ highlighting that cohabitants of index patients with mycobacterial infections represent an important sub-population for targeted public health interventions.

Current literature suggests that the first 2 years after the diagnosis of tuberculosis in the index patient are the most critical for tuberculosis detection among household contacts.^5^, ^6^ However, our study demonstrates a prolonged risk (extending to approximately 12 years) for tuberculosis, especially among adolescents and adults, suggesting potential deficits in tuberculosis screening and tuberculosis preventive treatment among these age groups. It is also possible that cohabitants with late-onset disease represent cases of LTBI reactivation or may be re-exposed to tuberculosis in or outside the home.^24^ In addition, some families had more than one cohabitant diagnosed with tuberculosis, which could have led to increased exposure to tuberculosis among other household members.

In our study, although children younger than 5 years did not represent the age group at highest risk for tuberculosis among contacts as found in previous studies,^24^, ^25^ they still presented a higher risk of developing tuberculosis after exposure to a patient with tuberculosis in their households when compared with the same age group in the 100MCohort. The observation that cohabitants younger than 5 years were diagnosed more quickly following the index patient's diagnosis than other age groups aligns with the evidence from a systematic review that demonstrates that among all contacts younger than five years who developed tuberculosis, 83% were diagnosed in the first 90 days of screening.^16^ Against our results we can speculate that children from this age group might have been detected and likely to be starting tuberculosis preventive treatment earlier, as this has been shown to reduce the risk of developing active tuberculosis among household's contacts.^8^, ^16^ However, these results must be interpreted with caution. First, the diagnosis of tuberculosis is more challenging among children.^16^ We found a lower percentage of contacts younger than 5 years detected with tuberculosis (1·7%) than the evidence from a systematic review and meta-analysis carried out in LMICs (6·8%).^2^ Second, the unavailability of tuberculosis preventive treatment and LTBI data for this study makes it impossible to confirm whether these children started tuberculosis preventive treatment.

We also found a higher risk of tuberculosis for contacts with index patients who had pulmonary tuberculosis or index patients who were younger than 5 years. These groups are already prioritised by the contact tracing policy in Brazil due to the importance of pulmonary tuberculosis in the transmission of the disease,^5^, ^19^, ^24^, ^25^ and due to the close contact and high likelihood of transmission between adult caregivers and children within families.^13^, ^26^ We can also speculate that the identification of an index patient younger than 5 years might trigger a more thorough search for a source patient (ie, the person who transmitted the disease) by the health services. Therefore, improving the investigation of contacts among index patients of all ages could further improve tuberculosis prevention and care in Brazil. It is also important to note that by the time the index patient in the current analysis was diagnosed, there might have been previous tuberculosis patients in a given household.

We found a higher tuberculosis incidence among historically disadvantaged racial or ethnical groups (ie, individuals who self-identified as being of Black, Pardo, or indigenous race or ethnicity), and those living in poor housing conditions. Therefore, our findings suggest that people living in socially and economically vulnerable contexts should be prioritised in the Brazilian contact tracing policy.

Finally, the lower municipal performance on tuberculosis indicators is associated with tuberculosis among contacts younger than 5 years and cohabitants detected more than 3 months after the index patient. This highlights the predominance of passive, non-systematic contact tracing in Brazil, which relies on index patients' voluntary indication of cohabitants for screening.^27^ Furthermore, the overload and high turnover of professionals in primary health care and the Family Health Strategy have been identified as barriers to effective contact tracing.^27^ The health services should therefore be strengthened in an active way, including systematic efforts to enhance screening of cohabitants and provide increased tuberculosis preventive treatment, thus reducing transmission within households and communities.^28^, ^29^, ^30^

This study provides unique insights into our understanding of tuberculosis in the LMIC context by looking at a population with high social vulnerability in a middle-income country with a high tuberculosis burden. Nevertheless, our study has some limitations. First, the 100MCohort represents only the lowest-income part of the Brazilian population and cannot be generalised to the whole population of Brazil. Second, as the households' composition was evaluated at the beginning of the follow-up, it is likely that the families' structures will have changed over the duration of follow-up. Third, this study used administrative data which are subject to incompleteness. Although CadÚnico is a social registry with reasonably good completeness,^10^ SINAN-TB is a surveillance system, which is subject to underreporting and preferential notification of severe tuberculosis, especially in the low-income areas of Brazil. However, our linkage with tuberculosis records achieved high accuracy, with a sensitivity of 94·6% and specificity of 93·6%. Therefore, more complete data would probably have led to more linked records and a higher tuberculosis incidence among household contacts. Fourth, as stated previously, variables such as intensity of exposure, LTBI testing, tuberculosis preventive treatment, and other key risk factors for tuberculosis disease (HIV or acquired immunodeficiency syndrome or immunosuppression, alcohol use, smoking, drug abuse, diabetes, undernutrition, and air pollution, among others) were not measured. Although we do not have reasons to believe in an imbalance in the distribution of those variables in household contacts and non-contacts, it is reasonable to assume that tuberculosis preventive treatment could be more frequently used in areas of better municipal performance of tuberculosis indicators. Finally, some populations (people experiencing homelessness, people deprived of their liberty, and people residing in long-stay institutions) considered at higher risk for tuberculosis were not included in the study as the concept of household contacts was impossible to apply in such contexts. In conclusion, our study found that cohabitants of people with tuberculosis have a high and sustained risk of tuberculosis and, therefore, constitute a unique group to whom public health intervention should be targeted. Our findings provide evidence to suggest that contact tracing policies in Brazil and other high-burden countries with similar social contexts should be extended and strengthened systematically to expand tuberculosis preventive treatment not only for children, but for adolescents and young adults living in poorer socioeconomic circumstances. We suggest that this will promote equitable tuberculosis prevention and care in LMICs and help to achieve national and international targets for tuberculosis elimination.

## Data sharing

The relevant data are available in the manuscript and the appendix. Data that are not presented in the Article or appendix are available upon reasonable request from the corresponding author.

## Declaration of interests

We declare no competing interests.
