# Supplementary material for: Incidence and risk factors of tuberculosis among 420 854 household contacts of patients with tuberculosis in the 100 Million Brazilian Cohort (2004–18): a cohort study
Source: Lancet Infect Dis. 2024 Jan;24(1):46–56. doi: 10.1016/S1473-3099(23)00371-7 (PMC10733584; doi:10.1016/S1473-3099(23)00371-7)
Supplement: Supplementary appendix [file mmc1.pdf]

THE LANCET
Infectious Diseases

## Supplementary appendix

This appendix formed part of the original submission and has been peer reviewed. We post it as supplied by the authors.

Supplement to: Pinto PFPS, Teixeira CSS, Ichihara MY, et al. Incidence and risk factors of tuberculosis among 420 854 household contacts of patients with tuberculosis in the 100 Million Brazilian Cohort (2004–18): a cohort study. *Lancet Infect Dis* 2023; published online Aug 14. https://doi.org/10.1016/S1473-3099(23)00371-7.

# Incidence and risk factors for tuberculosis among 420,854 household contacts of tuberculosis patients in the 100 Million Brazilian Cohort (2004-2018): A cohort study

**Supplementary material**

**Table of contents**

1. Data sources ........................................................................................................ 2

2. Accuracy analysis.................................................................................................... 3

3. Incidence of tuberculosis in the overall population.................................................... 5

4. Incidence of tuberculosis among household contacts by years after the detection of the index patient.................................................................................................................. 6

5. Analysis of risk factors for tuberculosis among household contacts with non-co-prevalent TB 7

6. Incidence of tuberculosis among household contacts <5 years old.............................. 9

7. Analysis of risk factors for tuberculosis among household contacts <5 years old.................. 11

8. Analysis of risk factors for tuberculosis by performance of TB indicators in the index patient's municipality of residence ............................................................................ 15

9. Percent attributable risk.......................................................................................... 18

10. Intraclass Correlation Coefficients (ICC).................................................................. 18

## 1. Data sources

### The *Cadastro Único* (CadÚnico) database and the 100 Million Brazilian Cohort

The 100 Million Brazilian Cohort was based on the baseline information of families, from January 1, 2001 to December 31, 2018, who sought to benefit from the Brazilian government's social programs through registration in the Unified Registry for Social Programs (in Portuguese: *Cadastro Único para Programas Sociais* – CadÚnico). The CadÚnico is an administrative database, to which Brazilians aged 16 or over can apply by registering their personal information (age, sex, self-identified race/ethnicity, education and others) and household information (household density, structural characteristics of the residence and others), as long as they are within one of these categories: (i) belong to a family with a monthly per capita income of up to half a minimum wage; (ii) belong to a family with a total monthly income of up to three minimum wages; (iii) belong to a family with an income greater than three minimum wages, provided that the registration is linked to inclusion in social programs in the three spheres of government; (iv) are the only resident of the household, or; (v) are experiencing homelessness.

Upon registration, individuals receive a unique identifier code and are searched for socioeconomic characteristics. At the end of 2017, CadÚnico had approximately 131 million individuals on its register, which represents around 50% of the Brazilian population. It is a social tool that identifies and characterizes especially low-income families, allowing the government to know the socioeconomic aspects of the poorest and use it for the selection of social programs.[1,2]

In addition to the information from CadÚnico, the Cohort is also composed of health-related databases. For our study, the information used to identify tuberculosis (TB) comes from the National System of Disease Notification (in Portuguese: *Sistema de Informação de Agravos de Notificação* - SINAN) and the Mortality Information System (in Portuguese: *Sistema de Informação sobre Mortalidade* - SIM).[3] Created by the Center for Integration of Data and Knowledge for Health/Oswaldo Cruz Foundation (CIDACS/FIOCRUZ),[2] the 100 Million Brazilian Cohort aims to facilitate research and continuous assessment of social determinants and the effects of social policies and programs in health contexts in Brazil. It has 246 variables with demographic and socioeconomic information at the individual and family level. The codes and linking algorithms between the databases were built to make efficient and specific links through five identifiers: the date of birth, the municipality of residence, the sex, the name and the mother's name of each individual presented in each of the databases.[2,4,5] The linkage was performed at the individual level in two-steps using the CIDACS-RL (Centro de Integração de Dados e Conhecimentos para Saúde – Record Linkage) (https://gitHub.com/gcgbarbosa/cidacs-rl). First, the inputs were linked deterministically. In the second step, for cases that were not deterministically linked, they were linked based on a similarity score for all pairwise comparisons, ranging from 0 to 1. The entries with the highest similarity scores were considered as linked pairs. The quality of each link for all causes between CadÚnico, SINAN and SIM has been extensively evaluated and validated.[6,7,8]

### REFERENCES


1. Sanni Ali M, Ichihara MY, Lopes LC, et al. Administrative data linkage in Brazil: Potentials for health technology assessment. Front Pharmacol 2019;**10**(SEP):1–20.
2. Barreto ML, Ichihara MY, Pescarini JM, et al. Cohort Profile: The 100 Million Brazilian Cohort. Int J Epidemiol 2022; 51: e27–38.
3. DATASUS. Ministério da Saúde [Internet]. [cited 2022 Dec 20]; Available from: https://datasus.saude.gov.br/
4. Pita R, Pinto C, Sena S, et al. On the Accuracy and Scalability of Probabilistic Data Linkage over the Brazilian 114 Million Cohort. IEEE J Biomed Heal Informatics 2018;**22**(2): 346–53.
5. Pinto C, Pita R, Barbosa G, et al. Probabilistic Integration of Large Brazilian Socioeconomic and Clinical Databases. Proc - IEEE Symp Comput Med Syst 2017;2017–June:515–20.
6. Barreto, Marcos1, Alves, André, Sena, Samila, Fiaccone, Rosemeire, Amorim, Leila, Ichihara, Maria Yuri, and Barreto M. Assessing the accuracy of probabilistic record linkage of social and health databases in the 100 million Brazilian cohort. Int J Popul Data Sci 2017; 2017;**1**(1):276
7. Pita R, Pinto C, Barreto M, et al. Design and evaluation of probabilistic record linkage methods supporting the Brazilian 100-million cohort initiative. Int J Popul Data Sci 2017;**1**(1):23889.
8. Barbosa GCG, Ali MS, Araujo B, et al. CIDACS-RL: a novel indexing search and scoring-based record linkage system for huge datasets with high accuracy and scalability. BMC Med Inform Decis Mak 2020; **20**:289.

## 2. Accuracy analysis

The accuracy analysis has as main objective to evaluate the quality of the non-deterministic linkage, regarding the similarity between the records. This step is done after finalizing and validating the linkage. It consists of obtaining a sample of records stratified by three ranges of score - similarity index (high score - above 0·95, intermediate score - values between 0·90 and 0·95, and low score - below 0·90). Relevant information is that, as of April 2021, records whose scores were greater than or equal to 0·95 and less than 1 started to compose the high score range. Pairs of records are evaluated manually and classified as pairs true or false pairs. Then, the Receiver Operating Characteristic (ROC) curve is constructed to define the best cut-off point, balancing between the best values of sensitivity (defined as the proportion of true pairs among those classified positively in the manual verification) and specificity (defined as the proportion of false pairs classified as such in manual verification). Thus, records with a score greater than or equal to the cut-off point are classified as linked, and those below the cut-off point are classified as unlinked.

The cases in which sensitivity values are set to 100% simply mean that in the sample all pairs of records classified as true by manual verification had score values greater than or equal to the best cut-off point, defined in the ROC curve.

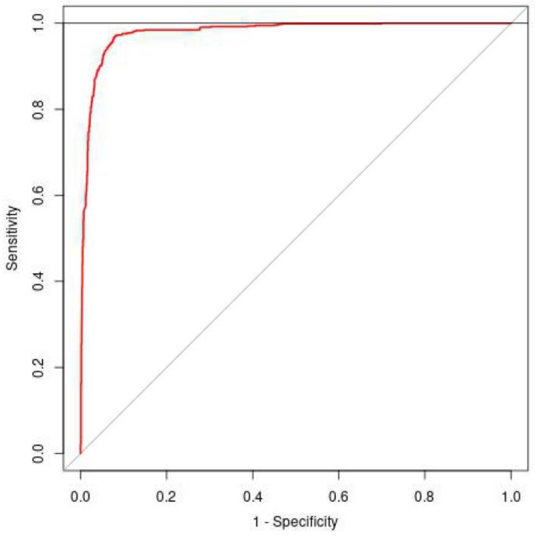


*Area under the curve: 0·9796 (95%CI:0·9740-0·9851)

**Figure S1. The 100 Million Brazilian Cohort x SINAN-TB linkage ROC curve, 2004-2019**

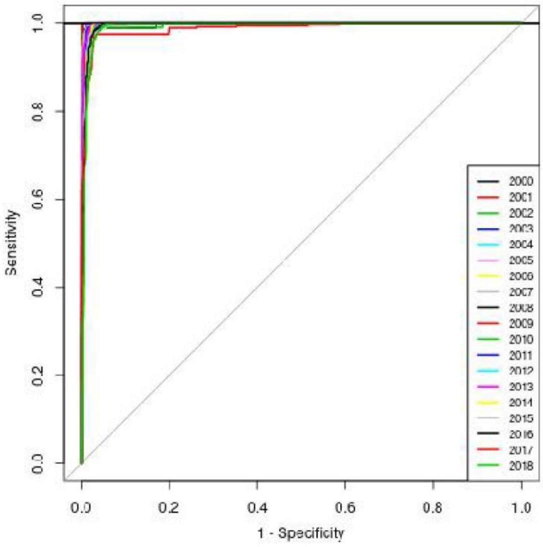


**Figure S2. The 100 Million Brazilian Cohort x SIM linkage ROC curve, 2000-2018**

**Table S1. The 100 Million Brazilian Cohort x SIM linkage area under the curve and 95% confidence intervals, 2000-2018**

| Year | Area under the curve | 95%CI |
|---|---|---|
| 2000 | 0·9990 | 0·9975-0·9990 |
| 2001 | 0·9996 | 0·9990-1·0000 |
| 2002 | 0·9983 | 0·9966-1·0000 |
| 2003 | 0·9989 | 0·9981-0·9998 |
| 2004 | 0·9990 | 0·9982-0·9997 |
| 2005 | 0·9986 | 0·9976-0·9996 |
| 2006 | 0·9967 | 0·9948-0·9986 |
| 2007 | 0·9994 | 0·9989-0·9999 |
| 2008 | 0·9960 | 0·9934-0·9987 |
| 2009 | 0·9973 | 0·9958-0·9988 |
| 2010 | 0·9959 | 0·9933-0·9985 |
| 2011 | 0·9975 | 0·9962-0·9989 |
| 2012 | 0·9959 | 0·9940-0·9978 |
| 2013 | 0·9988 | 0·9979-0·9996 |
| 2014 | 0·9948 | 0·9925-0·9971 |
| 2015 | 0·9960 | 0·9941-0·9978 |
| 2016 | 0·9949 | 0·9928-0·9971 |
| 2017 | 0·9883 | 0·9832-0·9934 |
| 2018 | 0·9920 | 0·9889-0·9951 |

CI-Confidence Interval

## 3. Incidence of tuberculosis in the overall population

**Table S2. Incidence of tuberculosis in the 100 Million Brazilian Cohort (overall population), 2004-2018**

| Characteristics | TB patients N (%) | Persons-years at risk | Incidence, for 100,000 person-years (95% CI) |
|---|---|---|---|
| Total | 168,804 (100·0) | 643,800,000 | 26·22 (26·10-26·35) |
| Sex | | | |
| Female | 71,739 (42·5) | 346,400,000 | 20·71 (20·56-20·86) |
| Male | 97,065 (57·5) | 297,400,000 | 32·64 (32·43-32·84) |
| Age (years) | | | |
| <5 | 6,875 (4·1) | 167,600,000 | 4·10 (4·01-4·20) |
| 5–14 | 25,395 (15·0) | 138,700,000 | 18·30 (18·08-18·53) |
| 15-19 | 16,182 (9·6) | 43,906,769 | 36·86 (36·29-37·42) |
| 20–59 | 108,236 (64·1) | 264,100,000 | 40·98 (40·74-41·23) |
| >60 | 12,116 (7·2) | 29,445,985 | 41·15 (40·42-41·89) |
| Race/ethnicity | | | |
| White | 43,913 (26·0) | 204,500,000 | 21·47 (21·27-21·67) |
| Black | 20,711 (12·3) | 43,220,896 | 47·92 (47·27-48·58) |
| Asian | 519 (0·31) | 2,235,212·2 | 23·22 (21·30-25·30) |
| Pardo | 95,339 (56·5) | 373,800,000 | 25·51 (25·34-25·67) |
| Indigenous | 2,457 (1·5) | 4,353,413·2 | 56·44 (54·25-58·71) |
| Education (years of study) | | | |
| Illiterate | 22,187 (13·1) | 98,253,862 | 22·58 (22·29-22·88) |
| <4 | 56,733 (33·6) | 200,700,000 | 28·26 (28·03-28·50) |
| 4-9 | 49,354 (29·2) | 164,900,000 | 29·93 (29·67-30·20) |
| >9 | 24,111 (14·3) | 108,100,000 | 22·30 (22·02-22·59) |
| Area of residence | | | |
| Urban | 20,660 (12·2) | 144,600,000 | 14·29 (14·09-14·48) |
| Rural | 146,010 (86·5) | 146,010 | 29·44 (29·30-29·59) |
| Brazilian region | | | |
| North | 21,114 (12·5) | 7,815,5845 | 27·01 (26·65-27·38) |
| North East | 49,584 (29·4) | 227,000,000 | 21·84 (21·65-22·04) |
| Midwest | 8,051 (4·8) | 49,315,581 | 16·32 (15·97-16·69) |
| South East | 72,256 (42·8) | 220,700,000 | 32·75 (32·51-32·99) |
| South | 17,793 (10·5) | 68,636,164 | 25·92 (25·54-26·31) |

CI-Confidence Interval

## 4. Incidence of tuberculosis among household contacts by years after the detection of the index patient

**Table S3. Incidence of tuberculosis among household contacts, The 100 Million Brazilian Cohort by years after the detection of the index patient, 2004-2018**

| Years | TB patients N (%) | Persons-years at risk | Incidence, for 100,000 person-years (95% CI) |
|---|---|---|---|
| 0-1 | 3,723 (41·58) | 390538·3 | 953·30 (923·16-984·42) |
| 1-2 | 1,468 (16·40) | 336512·2 | 436·24 (414·49-459·14) |
| 2-3 | 1,040 (11·62) | 288821·6 | 360·08 (338·85-383·65) |
| 3-4 | 760 (8·49) | 245557·7 | 309·50 (288·26-332·30) |
| 4-5 | 585 (6·53) | 206115·2 | 283·82 (261·73-307·78) |
| 5-6 | 410 (4·58) | 170385·3 | 240·63 (218·43-265·09) |
| 6-7 | 322 (3·60) | 1379967·0 | 233·34 (209·19-260·27) |
| 7-8 | 222 (2·48) | 108515·0 | 204·58 (179·36-233·34) |
| 8-9 | 172 (1·92) | 81884·8 | 210·05 (180·89-243·91) |
| 9-10 | 115 (1·28) | 58269·0 | 197·36 (164·39-236·94) |
| 10-11 | 82 (0·92) | 37255·3 | 220·10 (177·26-273·29) |
| 11-12 | 33 (0·37) | 19856·6 | 166·20 (188·15-233·77) |
| 12-13 | 17 (0·29) | 7692·5 | 220·99 (137·38-355·49) |
| 13-14 | 1 (0·01) | 2735·7 | 36·55 (5·15-259·49) |
| >14 | 3 (0·03) | 414·4 | 273·93 (233·48-2244·59) |
| Total | 8,953 (100·0) | 2092550·5 | 427·85 (419·08-436·81) |

CI-Confidence Interval

## 5. Analysis of risk factors for tuberculosis among household contacts with non-co-prevalent TB

**Table S4. Adjusted multilevel Poisson regression analysis of the association of distal, intermediate and proximal variables with TB among household contacts with non-co-prevalent TB, The 100 Million Brazilian Cohort, 2004-2018**

| | RR adjusted (CI 95%) n=348,567 |
|---|---|
| *Distal variables*[1] | |
| Area of residence | |
| Rural | 1 |
| Urban | 1·01 (0·91-1·11) |
| Brazilian region | |
| North | 1 |
| Northeast | 0·82 (0·72-0·93) |
| Midwest | 0·89 (0·73-1·08) |
| Southeast | 1·19 (1·05-1·34) |
| South | 1·16 (1·00-1·35) |
| Performance of TB indicators in the index patient's municipality [2] | |
| A (highest quality performance) | 1 |
| B (medium quality performance) | 0·93 (0·85-1·02) |
| C (lowest quality performance) | 1·11 (1·00-1·21) |
| | |
| *Intermediate variables*[3] | |
| Education (years of study) | |
| >9 | 1 |
| 4-9 | 1·09 (1·00-1·19) |
| <4 | 1·10 (1·00-1·21) |
| Illiterate | 1·04 (0·93-1·16) |
| Race/ethnicity | |
| White | 1 |
| Asian | 0·73 (0·38-1·40) |
| Pardo | 1·13 (1·05-1·21) |
| Black | 1·36 (1·24-1·50) |
| Indigenous | 2·03 (1·60-2·56) |
| House material | |
| Brick | 1 |
| Wood | 1·08 (0·97-1·19) |
| Other materials (taipa[4]) | 1·01 (0·90-1·14) |
| Water supply | |
| Public network | 1 |
| Well, spring or cistern | 0·91 (0·84-1·00) |
| Sewage | |
| Public network | 1 |
| Septic tank | 1·01 (0·91-1·12) |
| Rudimentary cesspit/ditch or another | 1·06 (0·98-1·14) |
| Lighting | |
| Electricity with household meter | 1 |
| Electricity with collective meter (one meter for multiple households) | 1·04 (0·92-1·17) |
| Electricity: without meter (irregular source) | 1·11 (1·02-1·23) |
| No electricity (e.g., lamp or candle) | 1·16 (1·04-1·30) |
| Garbage disposal | |
| Public collection | 1 |
| Burned, buried or another | 1·01 (0·91-1·13) |
| Household density (residents per room) | |
| <3 | 1 |
| ≥3 | 0·97 (0·89-1·06) |
| | |
| *Proximal variables*[5] | |
| Sex of the subsequent patient among contacts | |
| Female | 1 |
| Male | 1·25 (1·18-1·32) |
| Age (years) of the subsequent patient among contacts | |
| <5 | 1 |
| 5-14 | 1·46 (1·26-1·71) |
| 15-19 | 3·10 (2·65-3·61) |
| 20-59 | 2·32 (2·00-2·70) |
| >60 | 1·54 (1·26-1·90) |
| Sex of the index patient | |
| Male | 1 |

| | |
|---|---|
| Female | 1·32 (1·25-1·41) |
| Age (years) of the index patient | |
| >60 | 1 |
| 20–59 | 1·48 (1·28-1·71) |
| 15–19 | 2·31 (1·97-2·32) |
| 5–14 | 2·49 (2·06-3·03) |
| <5 | 3·24 (2·43-4·32) |
| Clinical classification of TB index patients | |
| Extrapulmonary | 1 |
| Pulmonary | 2·77 (2·45-3·13) |

1- Distal model (n=348,567) risk ratio (RR) was adjusted for area of residence and Brazilian region , and performance of TB indicators in the index patient's municipality of residence(distal variables).
2- Group A: represents municipalities with the highest quality performance for TB indicators; Group B: represents municipalities with medium quality performance for TB indicators; Group C: represents municipalities with the lowest quality performance for TB indicators
3- Intermediate model (n=348,567) RR was adjusted for education, race/ethnicity, house material, water supply, sewage, lighting, garbage disposal, household density (intermediate variables) plus area of residence and Brazilian region, and performance of TB indicators in the index patient's municipality of residence (distal variables).
4- Taipa is a construction method that consists of using clay and wood to build houses
5- Proximal model (n=348,567) RR was adjusted for sex and age of subsequent TB patient among contacts age, sex and age of TB index patient and clinical classification of TB index patient (proximal variables), plus education, race/ethnicity, house material, water supply, sewage, electricity supply, garbage disposal, household density (intermediate variables), plus area of residence and Brazilian region , and performance of TB indicators in the index patient's municipality of residence (distal variables).

## 6. Incidence of tuberculosis among household contacts <5 years old

**Table S5**. **Incidence of tuberculosis among household contacts under five years old by socioeconomic, geographic, household and TB index patient characteristics, The 100 Million Brazilian Cohort, 2004-2018**

| Characteristics | TB patients among contacts | Persons-years at risk | Incidence, for 100,000 persons-years (95% CI) |
|---|---|---|---|
| | N (%) | | |
| Total | 536 (100·0) | 211,017·2 | 254·01 (233·39-276·45) |
| Sex | | | |
| Female | 265 (49·4) | 104,468·2 | 253·66 (224·89-286·12) |
| Male | 271 (50·6) | 106,549·0 | 254·34 (225·79-286·50) |
| Missing | - | - | - |
| Age (years) | | | |
| <1 | 44 (8·2) | 12,680·8 | 346·98 (258·22-466·26) |
| 1 | 105 (19·6) | 32,590·8 | 322·18 (266·09-390·09) |
| 2 | 119 (22·2) | 47,087·8 | 252·72 (211·16-302·46) |
| 3 | 127 (23·7) | 55,611·3 | 228·37 (191·92-271·75) |
| 4 | 141 (26·3) | 63,046·6 | 223·64 (189·61-263·78) |
| Missing | - | - | - |
| Race/ethnicity | | | |
| White/Asian | 166 (31·0) | 60,615·9 | 273·86 (235·21-318·85) |
| Black | 41 (7·6) | 14,954·2 | 274·17 (201·88-372·35) |
| Pardo | 296 (55·2) | 129,854·2 | 227·95 (203·40-255·45) |
| Indigenous | 33 (6·2) | 5,447·3 | 605·80 (430·68-852·13) |
| Missing | 11 (2·1) | - | - |
| Education (years of study) | | | |
| >9 | 80 (16·3) | 24,953·0 | 320·60 (257·51-399·15) |
| 4-9 | 170 (34·7) | 64,921·1 | 261·86 (225·31-304·33) |
| <4 | 179 (36·5) | 70,576·6 | 253·62 (219·06-293·64) |
| Illiterate | 61 (12·4) | 28,684·1 | 212·66 (165·46-273·32) |
| Missing | 46 (8·6) | - | - |
| Area of residence | | | |
| Urban | 68 (12·7) | 30,265·1 | 224·68 (177·15-284·96) |
| Rural | 467 (87·1) | 180,733·6 | 258·39 (235·99-282·92) |
| Missing | 1 (0·2) | - | - |
| Brazilian region | | | |
| North | 57 (10·6) | 28,911·8 | 197·07 (152·07-255·59) |
| North East | 108 (20·1) | 67,624·9 | 160·56 (132·96-193·88) |
| Midwest | 31 (5·8) | 9,598·5 | 322·97 (227·13-459·24) |
| South East | 282 (52·6) | 83,346·6 | 338·35 (301·07-380·23) |
| South | 58 (10·8) | 21,895·3 | 264·90 (204·79-342·65) |
| Missing | - | - | - |
| Household density (residents per room) | | | |
| <3.0 | 446 (85·6) | 183,420·1 | 243·16 (221·61-266·80) |
| ≥3.0 | 75 (14·4) | 25,122·1 | 298·54 (238·08-374·36) |
| Missing | 15 (2·8) | - | - |
| House material | | | |
| Brickwork | 389 (74·7) | 155,093·8 | 250·82 (227·10-277·02) |
| Wood | 93 (17·9) | 31,915·7 | 291·39 (237·80-357·06) |
| Another materials (taipa[1]) | 39 (7·5) | 21,532·7 | 181·12 (132·33-247·89) |
| Missing | 15 (2·8) | - | - |
| Water supply | | | |
| Public network | 401 (77·0) | 154,735·4 | 259·15 (234·99-285·80) |
| Well, spring or cistern | 120 (23·0) | 53,806·8 | 223·02 (186·48-266·71) |
| Missing | 15 (2·8) | - | - |
| Sewage | | | |
| Public network | 294 (58·0) | 107,218·9 | 274·20 (244·59-307·41) |
| Septic tank | 56 (11·0) | 25,688·9 | 217·99 (167·76-283·26) |
| Rudimentary cesspit/ditch or another | 157 (31·0) | 72,175·6 | 217·52 (186·03-254·36) |
| Missing | 29 (5·4) | - | - |
| Lighting | | | |
| Electricity with household meter | 330 (63·3) | 145,834·6 | 226·28 (203·14-252·06) |
| Electricity with collective meter (one meter for multiple households): | 57 (10·9) | 15,469·6 | 368·46 (284·22-477·68) |
| Electricity without meter (irregular source) | 77 (14·8) | 27,275·0 | 282·31 (225·80-352·96) |
| No electricity (e.g., lamp, candle or another | 57 (10·9) | 19,963·0 | 285·53 (220·24-370·16) |
| Missing | 15 (2·8) | - | - |
| Garbage disposal | | | |
| Public collection | 445 (85·4) | 169,784·6 | 262·10 (238·84-287·62) |
| Burned, buried, another | 76 (14·6) | 38,757·6 | 196·09 (156·61-245·53) |
| Missing | 15 (2·8) | - | - |

| | | | |
|---|---|---|---|
| Performance of TB indicators in the index patient's municipality [2] | | | |
| A (highest quality performance) | 161 (31·3) | 69,093·2 | 233·02 (199·67-271·94) |
| B (medium quality performance) | 120 (23·3) | 49,300·5 | 243·40 (203·53-291·09) |
| C (lowest quality performance) | 234 (45·4) | 81,945·5 | 285·56 (251·22-324·59) |
| Missing | 21 (3·9) | - | - |
| Sex of the TB index patient | | | |
| Female | 371 (69·2) | 117,888·9 | 314·70 (284·26-348·41) |
| Male | 165 (30·8) | 93,128·3 | 177·17 (152·10-206·38) |
| Missing | - | - | - |
| Age (years) of the TB index patient | | | |
| <5 | 34 (6·8) | 4,122·6 | 824·73 (589·29-1154·23) |
| 5-14 | 36 (7·2) | 8,681·7 | 414·66 (299·11-574·86) |
| 15-19 | 39 (7·8) | 11,720·8 | 332·74 (243·11-455·42) |
| 20-49 | 416 (82·9) | 170,657·5 | 243·76 (221·43-268·35) |
| ≥50 | 11 (2·2) | 15,834·6 | 69·47 (38·47-125·44) |
| Missing | - | - | - |
| Clinical classification of the TB index patient | | | |
| Extrapulmonary | 23 (4·3) | 25,783·3 | 89·20 (59·28-134·24) |
| Pulmonary | 513 (95·7) | 185,233·9 | 276·95 (253·99-301·98) |
| Missing | - | - | - |

1- Taipa is a construction method that consists of using clay and wood to build houses

2- Group A: represents municipalities with the highest quality performance for TB indicators; Group B: represents municipalities with medium quality performance for TB indicators; Group C: represents municipalities with the lowest quality performance for TB indicators

## 7. Analysis of risk factors for tuberculosis among household contacts <5 years old

**Table S6. Adjusted Poisson regression analysis of the association of sociodemographic, geographic, household factors and TB index patient characteristics (distal, intermediate and proximal variables) with TB among household contacts under five years old with co-prevalent and non-co-prevalent TB, The 100 Million Brazilian Cohort, 2004-2018**

| | **RR adjusted (CI 95%) n=26,651** |
|---|---|
| *Distal variables*[1] | |
| Area of residence | |
| Rural | 1 |
| Urban | 1·19 (0·85-1·68) |
| Brazilian region | |
| North | 1 |
| Northeast | 0·83 (0·58-1·18) |
| Midwest | 1·57 (0·94-2·61) |
| Southeast | 1·71 (1·25-2·36) |
| South | 1·19 (0·78-1·81) |
| Performance of TB indicators in the index patient's municipality [2] | |
| A (highest quality performance) | 1 |
| B (medium quality performance) | 1·23 (0·95-1·58) |
| C (lowest quality performance) | 1·29 (1·04-1·61) |
| | |
| *Intermediate variables*[3] | |
| Education (years of study) | |
| >9 | 1 |
| 4-9 | 0·90 (0·68-1·19) |
| <4 | 0·84 (0·63-1·12) |
| Illiterate | 0·82 (0·57-1·17) |
| Race/ethnicity | |
| White/Asian | 1 |
| Pardo | 1·05 (0·84-1·31) |
| Black | 1·11 (0·76-1·60) |
| Indigenous | 3·03 (1·67-5·51) |
| House material | |
| Brick | 1 |
| Wood | 1·53 (1·13-2·07) |
| Other materials (taipa[4]) | 0·91 (0·59-1·42) |
| Water supply | |
| Public network | 1 |
| Well, spring or cistern | 0·92 (0·70-1·21) |
| Sewage | |
| Public network | 1 |
| Septic tank | 1·27 (0·92-1·75) |
| Rudimentary cesspit/ditch or another | 1·04 (0·80-1·35) |
| Lighting | |
| Electricity with household meter | 1 |
| Electricity with collective meter (one meter for multiple households) | 1·31 (0·96-1·77) |
| Electricity without meter (irregular source) | 1·00 (0·76-1·33) |
| No electricity (e.g., lamp or candle) | 1·14 (0·80-1·64) |
| Garbage disposal | |
| Public collection | 1 |
| Burned, buried or another | 0·86 (0·59-1·25) |
| Household density (residents per room) | |
| <3 | 1 |
| ≥3 | 1·17 (0·89-1·54) |
| | |
| *Proximal variables*[5] | |
| Sex of the subsequent TB patient among contacts | |
| Female | 1 |
| Male | 0·99 (0·82-1·19) |
| Age (years) of the subsequent TB patient among contacts | |
| <1 | 1 |
| 1–1y11m29d | 0·84 (0·57-1·24) |
| 2–2y11m29d | 0·62 (0·42-0·91) |
| 3–3y11m29d | 0·52 (0·35-0·75) |
| 4-4y11m29d | 0·48 (0·33-0·69) |
| Sex of the index patient | |
| Male | 1 |

| | |
|---|---|
| Female | 1·79 (1·46-2·19) |
| Age (years) of the index patient | |
| >50 | 1 |
| 20–49 | 2·98 (1·58-5·61) |
| 15–19 | 3·48 (1·70-7·09) |
| 5–14 | 5·47 (2·65-11·28) |
| <5 | 11·07 (5·34-22·98) |
| Clinical classification of TB index patients | |
| Extrapulmonary | 1 |
| Pulmonary | 3·44 (2·17-5·46) |

1- Distal model (n=26,651) risk ratio (RR) was adjusted for area of residence and Brazilian region, and municipality performance of TB indicators (distal variables).

2- Group A: represents municipalities with the highest quality performance for TB indicators; Group B: represents municipalities with medium quality performance for TB indicators; Group C: represents municipalities with the lowest quality performance for TB indicators 3- Intermediate model (n=26,651) RR was adjusted for education, race/ethnicity, house material, water supply, sewage, lighting, garbage, household density (intermediate variables) plus area and Brazilian region of residence, and performance of TB indicators in the index patient's municipality of residence (distal variables).

4- Taipa is a construction method that consists of using clay and wood to build houses

5- Proximal model (n=26,651) RR was adjusted for sex and age of subsequent TB patient among contacts age, sex and age of TB index patient and clinical classification of the TB index patient (proximal variables), plus education, self-identified race/ethnicity, house material, water supply, sewage, lighting, garbage disposal, household density (intermediate variables), plus area of residence and Brazilian region, and performance of TB indicators in the index patient's municipality of residence (distal variables).

**Table S7. Adjusted Poisson regression analysis of the association of sociodemographic, geographic, household factors and TB index patient characteristics (distal, intermediate and proximal variables) with TB among household contacts under five years old with non-co-prevalent TB, The 100 Million Brazilian Cohort, 2004-2018**

| | **RR adjusted (CI 95%) n=26,403** |
|---|---|
| *Distal variables[1]* | |
| Area of residence | |
| Rural | 1 |
| Urban | 0·82 (0·54-1·24) |
| Brazilian region | |
| North | 1 |
| Northeast | 0·69(0·44-1·07) |
| Midwest | 1·16 (0·60-2·25) |
| Southeast | 1·18 (0·80-1·76) |
| South | 1·07 (0·63-1·82) |
| Performance of TB indicators in the index patient's municipality [2] | |
| A (highest quality performance) | 1 |
| B (medium quality performance) | 0·84 (0·57-1·23) |
| C (lowest quality performance) | 1·31 (0·97-1·77) |
| | |
| *Intermediate variables[3]* | |
| Education (years of study) | |
| >9 | 1 |
| 4-9 | 1·06 (0·67-1·69) |
| <4 | 1·21 (0·77-1·91) |
| Illiterate | 1·09 (0·62-1·92) |
| Race/ethnicity | |
| White/Asian | 1 |
| Pardo | 1·25 (0·87-1·79) |
| Black | 1·62 (0·96-2·74) |
| Indigenous | 2·08 (0·81-5·32) |
| House material | |
| Brick | 1 |
| Wood | 1·47 (0·94-2·30) |
| Other materials (taipa[4]) | 1·00 (0·55-1·83) |
| Water supply | |
| Public network | 1 |
| Well, spring or cistern | 0·79 (0·53-1·20) |
| Sewage | |
| Public network | 1 |
| Septic tank | 1·14 (0·69-1·87) |
| Rudimentary cesspit/ditch or another | 1·12 (0·76-1·64) |
| Lighting | |
| Electricity with household meter | 1 |
| Electricity: with collective meter (one meter for multiple households) | 1·24 (0·77-2·24) |
| Electricity without meter (irregular source) | 0·97 (0·64-1·47) |
| No electricity (e.g., lamp or candle) | 0·96 (0·56-1·67) |
| Garbage disposal | |
| Public collection | 1 |
| Burned, buried or another | 1·12 (0·66-1·87) |
| Household density (residents per room) | |
| <3 | 1 |
| ≥3 | 1·03 (0·67-1·57) |
| | |
| *Proximal variables[5]* | |
| Sex of the subsequent TB patient among contacts | |
| Female | 1 |
| Male | 0·79 (0·60-1·05) |
| Age (years) of the subsequent TB patient among contacts | |
| <1 | 1 |
| 1–1y11m29d | 0·58 (0·35-0·98) |
| 2–2y11m29d | 0·32 (0·19-0·55) |
| 3–3y11m29d | 0·38 (0·23-0·62) |
| 4-4y11m29d | 0·35 (0·22-0·58) |
| Sex of the index patient | |
| Male | 1 |
| Female | 1·69 (1·25-2·28) |
| Age (years) of the index patient | |

| | |
|---|---|
| >50 | 1 |
| 20–49 | 1·45 (0·73-2·88) |
| 15–19 | 2·45 (1·10-5·43) |
| 5–14 | 2·40 (1·00-5·75) |
| <5 | 4·35 (1·75-10·79) |
| Clinical classification of the TB index patients | |
| Extrapulmonary | 1 |
| Pulmonary | 1·90 (1·10-3·29) |

1- Distal model (n=26,403) risk ratio (RR) was adjusted for area of residence and Brazilian region, and performance of TB indicators in the index patient's municipality of residence (distal variables).

2- Group A: represents municipalities with the highest quality performance for TB indicators; Group B: represents municipalities with medium quality performance for TB indicators; Group C: represents municipalities with the lowest quality performance for TB indicators

3- Intermediate model (n=26,403) RR was adjusted for education, self-identified race/ethnicity, house material, water supply, sewage, lighting, garbage disposal, household density (intermediate variables) plus area of residence and Brazilian region, and municipality performance of TB indicators (distal variables).

4- Taipa is a construction method that consists of using clay and wood to build houses

5- Proximal model (n=26,403) RR was adjusted for sex and age of subsequent TB patient among contacts age, sex and age of TB index patient and clinical classification of the TB index patient (proximal variables), plus education, race/ethnicity, house material, water supply, sewage, electricity supply, garbage, household density (intermediate variables), plus area of residence and Brazilian region , and performance of TB indicators in the index patient's municipality of residence (distal variables).

## 8. Analysis of risk factors for tuberculosis by performance of TB indicators in the index patient's municipality of residence

**Table S8**. **Multilevel Poisson regression analysis adjusted of distal, intermediate and proximal variables with active TB among household contacts with co-prevalent and non-co-prevalent TB by performance of TB indicators in the index patient's municipality of residence, The 100 Million Brazilian Cohort, 2004-2018**

| | **Group A (Highest quality performance) n=117,550** | **Group B (Medium quality performance) n=79,448** | **Group C (Lowest quality performance) n=153,121** |
|---|---|---|---|
| | **RR adjusted (CI 95%)** | **RR adjusted (CI 95%)** | **RR adjusted (CI 95%)** |
| *Distal variables*[1] | | | |
| Area of residence | | | |
| Rural | 1 | 1 | 1 |
| Urban | 1·05 (0·90-1·22) | 1·13 (0·97-1·33) | 0·96 (0·80-1·14) |
| Brazilian region | | | |
| North | 1 | 1 | 1 |
| Northeast | 0·74 (0·60-0·91) | 0·77 (0·61-0·98) | 0·87 (0·70-1·07) |
| Midwest | 1·09 (0·83-1·44) | 1·04 (0·71-1·53) | 0·98 (0·71-1·36) |
| Southeast | 1·23 (1·01-1·49) | 1·09 (0·87-1·38) | 1·29 (1·06-1·58) |
| South | 1·12 (0·90-1·40) | 1·13 (0·86-1·49) | 1·38 (1·04-1·82) |
| *Intermediate variables*[2] | | | |
| Education (years of study) | | | |
| >9 | 1 | 1 | 1 |
| 4-9 | 1·00 (0·86-1·16) | 1·07 (0·88-1·28) | 1·03 (0·92-1·16) |
| <4 | 1·05 (0·91-1·22) | 0·93 (0·77-1·12) | 1·02 (0·91-1·15) |
| Illiterate | 1·02 (0·86-1·22) | 0·92 (0·73-1·15) | 0·92 (0·79-1·07) |
| Race/ethnicity | | | |
| White/Asian | 1 | 1 | 1 |
| Pardo | 1·13 (1·01-1·25) | 1·07 (0·93-1·24) | 1·12 (1·02-1·24) |
| Black | 1·32 (1·15-1·53) | 1·49 (1·22-1·81) | 1·16 (1·02-1·33) |
| Indigenous | 2·08 (1·50-2·87) | 2·28 (1·48-3·51) | 2·29 (1·59-3·31) |
| House material | | | |
| Brick | 1 | 1 | 1 |
| Wood | 1·12 (0·97-1·30) | 1·33 (1·09-1·61) | 1·02 (0·89-1·18) |
| Other materials (taipa[3]) | 1·06 (0·88-1·27) | 0·94 (0·75-1·17) | 0·93 (0·77-1·13) |
| Water supply | | | |
| Public network | 1 | 1 | 1 |
| Well, spring or cistern | 0·96 (0·83-1·11) | 0·88 (0·74-1·04) | 0·93 (0·83-1·04) |
| Sewage | | | |
| Public network | 1 | 1 | 1 |
| Septic tank | 0·97 (0·81-1·16) | 0·98 (0·81-1·19) | 1·02 (0·88-1·18) |
| Rudimentary cesspit/ditch or another | 1·03 (0·91-1·17) | 1·04 (0·88-1·22) | 1·05 (0·95-1·16) |
| Lighting | | | |
| Electricity with household meter | 1 | 1 | 1 |
| Electricity with collective meter | 1·05 (0·88-1·25) | 1·10 (0·87-1·37) | 1·10 (0·94-1·29) |
| Electricity without meter (irregular source) | 1·23 (1·07-1·42) | 1·32 (1·06-1·65) | 1·03 (0·91-1·15) |
| No electricity (e.g., lamp or candle) | 1·20 (1·01-1·44) | 1·21 (0·97-1·51) | 1·04 (0·89-1·22) |
| Garbage disposal | | | |
| Public collection | 1 | 1 | 1 |
| Burned, buried or another | 0·92 (0·77-1·09) | 1·03 (0·84-1·27) | 1·08 (0·93-1·27) |
| Household density (residents per room) | | | |
| <3 | 1 | 1 | 1 |
| ≥3 | 0·95 (0·82-1·10) | 0·80 (0·66-0·97) | 1·03 (0·92-1·15) |
| *Proximal variables*[4] | | | |
| Sex of the subsequent TB patient among contacts | | | |
| Female | 1 | 1 | 1 |
| Male | 1·26 (1·17-1·37) | 1·26 (1·13-1·40) | 1·21 (1·13-1·30) |
| Age (years) of the subsequent TB patient among contacts | | | |
| <5 | 1 | 1 | 1 |
| 5–14 | 0·84 (0·70-1·02) | 0·71 (0·57-0·89) | 0·88 (0·75-1·03) |
| 15-19 | 1·79 (1·47-2·18) | 1·29 (1·02-1·64) | 1·59 (1·34-1·88) |
| 20-59 | 1·48 (1·23-1·78) | 1·04 (0·84-1·29) | 1·21 (1·03-1·42) |
| ≥60 | 0·94 (0·71-1·26) | 0·88 (0·63-1·22) | 0·87 (0·67-1·11) |
| Sex of the index patient | | | |
| Male | 1 | 1 | 1 |
| Female | 1·41 (1·29-1·55) | 1·40 (1·25-1·58) | 1·37 (1·26-1·48) |
| Age (years) of the index patient | | | |
| >60 | 1 | 1 | 1 |

| | | | |
|---|---|---|---|
| 20–59 | 1·40 (1·11-1·75) | 1·54 (1·16-2·05) | 1·33 (1·09-1·63) |
| 15–19 | 2·33 (1·91-2·99) | 2·69 (1·95-3·70) | 1·86 (1·49-2·33) |
| 5–14 | 2·58 (1·93-3·45) | 3·00 (2·04-4·39) | 2·66 (2·06-3·44) |
| <5 | 3·89 (2·55-5·93) | 4·77 (2·80-8·14) | 4·04 (2·84-5·75) |
| Clinical classification of the TB index patients | | | |
| Extrapulmonary | 1 | 1 | 1 |
| Pulmonary | 2·40 (2·00-2·87) | 2·65 (2·09-3·36) | 3·37 (2·84-4·00) |

Group A: represents municipalities with the highest quality performance for TB indicators; Group B: represents municipalities with medium quality performance for TB indicators; Group C: represents municipalities with the lowest quality performance for TB indicators 1-Distal model: risk ratio (RR) was adjusted for area of residence and Brazilian region (distal variables).

2- Intermediate model: RR was adjusted for education, self-identified race/ethnicity, house material, water supply, sewage, lighting, garbage disposal, household density (intermediate variables) plus area of residence and Brazilian region (distal variables).

3- Taipa is a construction method that consists of using clay and wood to create houses

4- Proximal model: RR was adjusted for sex and age of subsequent TB patient among contacts age, sex and age of index TB patient and clinical classification of TB index patient (proximal variables), plus education, self-identified race/ethnicity, house material, water supply, sewage, lighting, garbage disposal, household density (intermediate variables), plus area of residence and Brazilian region (distal variables).

**Table S9. Adjusted multilevel Poisson regression analysis of distal, intermediate and proximal variables with active TB among household contacts with non-co-prevalent TB by performance of TB indicators in the index patient's municipality, The 100 Million Brazilian Cohort, 2004-2018**

| | Group A (Highest quality performance) n=117,038 | Group B (Medium quality performance) n=79,099 | Group C (Lowest quality performance) n=152,430 |
|---|---|---|---|
| | **RR adjusted (CI 95%)** | **RR adjusted (CI 95%)** | **RR adjusted (CI 95%)** |
| *Distal variables*[1] | | | |
| Area of residence | | | |
| Rural | 1 | 1 | 1 |
| Urban | 0·96 (0·82-1·12) | 1·11 (0·94-1·32) | 0·96 (0·80-1·16) |
| Brazilian region | | | |
| North | 1 | 1 | 1 |
| Northeast | 0·83 (0·67-1·02) | 0·80 (0·62-1·02) | 0·84 (0·68-1·05) |
| Midwest | 0·93 (0·69-1·25) | 0·85 (0·56-1·30) | 0·87 (0·62-1·21) |
| Southeast | 1·24 (1·01-1·52) | 1·15 (0·90-1·46) | 1·16 (0·95-1·42) |
| South | 1·04 (0·82-1·32) | 1·21 (0·90-1·61) | 1·34 (1·02-1·77) |
| *Intermediate variables*[2] | | | |
| Education (years of study) | | | |
| >9 | 1 | 1 | 1 |
| 4-9 | 1·07 (0·91-1·26) | 1·20 (0·96-1·49) | 1·07 (0·93-1·22) |
| <4 | 1·13 (0·96-1·33) | 1·11 (0·90-1·38) | 1·09 (0·95-1·24) |
| Illiterate | 1·10 (0·90-1·33) | 1·09 (0·85-1·41) | 0·98 (0·83-1·16) |
| Race/ethnicity | | | |
| White/Asian | 1 | 1 | 1 |
| Pardo | 1·16 (1·03-1·30) | 1·05 (0·891·23) | 1·14 (1·03-1·27) |
| Black | 1·46 (1·25-1·71) | 1·56 (1·26-1·93) | 1·23 (1·07-1·42) |
| Indigenous | 2·04 (1·43-2·91) | 2·34 (1·45-3·77) | 1·85 (1·21-2·81) |
| House material | | | |
| Brick | 1 | 1 | 1 |
| Wood | 1·08 (0·92-1·27) | 1·29 (1·04-1·61) | 0·97 (0·831·13) |
| Other materials (taipa[3]) | 1·12 (0·93-1·36) | 1·04 (0·82-1·32) | 0·90 (0·73-1·11) |
| Water supply | | | |
| Public network | 1 | 1 | 1 |
| Well, spring or cistern | 0·97 (0·83-1·13) | 0·89 (0·74-1·08) | 0·90 (0·79-1·01) |
| Sewage | | | |
| Public network | 1 | 1 | 1 |
| Septic tank | 1·01 (0·83-1·23) | 0·96 (0·77-1·19) | 1·04 (0·89-1·22) |
| Rudimentary cesspit/ditch or another | 1·07 (0·93-1·23) | 1·04 (0·87-1·24) | 1·06 (0·95-1·19) |
| Lighting | | | |
| Electricity with household meter | 1 | 1 | 1 |
| Electricity with collective meter (one meter for multiple households) | 1·01 (0·84-1·23) | 1·03 (0·80-1·33) | 1·06 (0·89-1·27) |
| Electricity without meter (irregular source) | 1·19 (1·02-1·39) | 1·31 (1·03-1·66) | 1·02 (0·90-1·16) |
| No electricity (e.g., lamp or candle) | 1·20 (0·99-1·45) | 1·15 (0·90-1·48) | 1·14 (0·96-1·35) |
| Garbage disposal | | | |
| Public collection | 1 | 1 | 1 |
| Burned, buried or another | 0·89 (0·74-1·08) | 0·96 (0·76-1·21) | 1·15(0·97-1·35) |
| Household density (residents per room) | | | |
| <3 | 1 | 1 | 1 |
| ≥3 | 0·95 (0·81-1·10) | 0·79 (0·640·98) | 1·06 (0·94-1·20) |
| *Proximal variables*[4] | | | |
| Sex of the subsequent TB patient among contacts | | | |
| Female | 1 | 1 | 1 |
| Male | 1·29 (1·18-1·41) | 1·25 (1·11-1·41) | 1·21 (1·12-1·31) |
| Age (years) of the subsequent TB patient among contacts | | | |
| <5 | 1 | 1 | 1 |
| 5–14 | 1·34 (1·03-1·73) | 1·62 (1·13-2·30) | 1·49 (1·19-1·87) |
| 15-19 | 3·17 (2·45-4·12) | 3·42 (2·38-4·89) | 2·91 (2·32-3·65) |
| 20-59 | 2·39 (1·86-3·08) | 2·54 (1·79-3·59) | 2·18 (1·75-2·72) |
| ≥60 | 1·38 (0·96-1·97) | 2·04 (1·31-3·19) | 1·46 (1·07-1·99) |
| Sex of the index patient | | | |
| Male | 1 | 1 | 1 |
| Female | 1·32 (1·20-1·46) | 1·30 (1·14-1·48) | 1·33 (1·22-1·45) |
| Age (years) of the index patient | | | |
| >60 | | 1 | 1 |
| 20–59 | 1·40 (1·09-1·79) | 1·80 (1·30-2·51) | 1·41 (1·13-1·76) |
| 15–19 | 2·34 (1·78-3·07) | 3·30 (2·29-4·75) | 1·99 (1·56-2·54) |
| 5–14 | 2·26 (1·64-3·12) | 3·02 (1·93-4·72) | 2·48 (1·86-3·3) |

| | | | |
|---|---|---|---|
| <5 | 3·13 (1·93-5·09) | 4·35 (2·25-8·41) | 2·94 (1·92-4·49) |
| Clinical classification of TB index patients | | | |
| Extrapulmonary | 1 | 1 | 1 |
| Pulmonary | 2·27 (1·87-2·75) | 2·65 (2·03-3·46) | 3·34 (2·76-4·04) |

Group A: represents municipalities with the highest quality performance for TB indicators; Group B: represents municipalities with medium quality performance for TB indicators; Group C: represents municipalities with the lowest quality performance for TB indicators 1-Distal model: risk ratio (RR) was adjusted for area of residence and Brazilian region (distal variables).
2- Intermediate model: RR was adjusted for education, self-identified race/ethnicity, house material, water supply, sewage, lighting, garbage disposal, household density (intermediate variables) plus area of residence and Brazilian region (distal variables).
3- Taipa is a construction method that consists of using clay and wood to create houses
4- Proximal model: RR was adjusted for sex and age of subsequent TB patient among contacts age, sex and age of the index TB patient and clinical classification of the TB index patient (proximal variables), plus education, self-identified race/ethnicity, house material, water supply, sewage, lighting, garbage disposal, household density (intermediate variables), plus area of residence and Brazilian region (distal variables).

## 9. Percent attributable risk

**Table S10. Percent attributable risk of having TB in exposed individuals, The 100 Million Brazilian Cohort, 2004-2018**

| Population | Incidence, for 100,000 person-years (95% CI) | $\%AR_{exp}$* |
|---|---|---|
| Overall population | 26·2 (26·1-26·4) | |
| Household contacts | 427·8 (419·1-436·8) | 93·9 |
| Overall population <5 years | 4·1 (4·0-4·2) | |
| Household contacts <5 years | 254·0 (233·4-276·4) | 98·4 |

*Percent attributable risk: $\%AR_{exp}=Incidence_{exp}-Incidence_{nexp}/Incidence_{exp} \times 100$

## 10. Intraclass Correlation Coefficients (ICC)

**Table S11. Incidence of tuberculosis among contacts by level and intraclass correlation coefficient (ICC) by each multilevel Poisson regression model performed, The 100 Million Brazilian Cohort, 2004-2018**

| Model | Incidence/100,000 person-years (95%CI) | % ICC (95% CI) |
|---|---|---|
| Including all contacts with co-prevalent and non-co-prevalent TB | | |
| Municipality-level | 150·8 (144·3-157·6) | 8·1 (4·7-13·7) |
| Household-level | 70·8 (66·8-75·0) | 42·9 (41·1-44·6) |
| Including only contacts with non-co-prevalent TB | | |
| Municipality-level | 119·7 (113·9-125·6) | 8·6 (4·9-15·3) |
| Household-level | 576·4 (540·1-615·1) | 43·6 (41·7-45·6) |
